# 2,8,9-Tris(2-methyl­prop­yl)-2,5,8,9-tetra­aza-1λ^5^-phosphatricyclo­[3.3.3.0^1,5^]undecan-5-ium chloride dihydrate

**DOI:** 10.1107/S1600536812045618

**Published:** 2012-11-10

**Authors:** Junseong Lee, Youngjo Kim

**Affiliations:** a Department of Chemistry, Chonnam National University, Gwangju 500-757, Republic of Korea; b Department of Chemistry, Chungbuk National University, Cheongju, Chungbuk 361-763, Republic of Korea

## Abstract

The asymmetric unit of the title hydrated salt, C_18_H_40_N_4_P^+^·Cl^−^·2H_2_O, consists of two ionic mol­ecules and four water mol­ecules. The mol­ecular geometry around the penta­coordinate P atom is trigonal–bipyramidal, with a H atom and an apical N atom in axial positions and three N atoms with isobutyl substituents in equatorial positions. The Cl^−^ ions and water mol­ecules are connected *via* O—H⋯Cl hydrogen bonds, forming chains along [100]. The ethyl­ene bridging groups are disordered with refined site-occupancy ratios of 0.578 (9):0.422 (9).

## Related literature

For background to the applications of related compounds, see: Raders & Verkade (2010 ▶); Tang *et al.* (1993 ▶); Verkade & Kisang (2003 ▶); Zhou *et al.* (2011 ▶). For similar structure types, see: Kingston & Verkade (2005 ▶); Kisanga & Verkade (2001 ▶); Liu *et al.* (1999 ▶, 2000 ▶); Mohan *et al.* (1996 ▶); Thirupathi *et al.* (2003 ▶); Wroblewski *et al.* (1995 ▶).
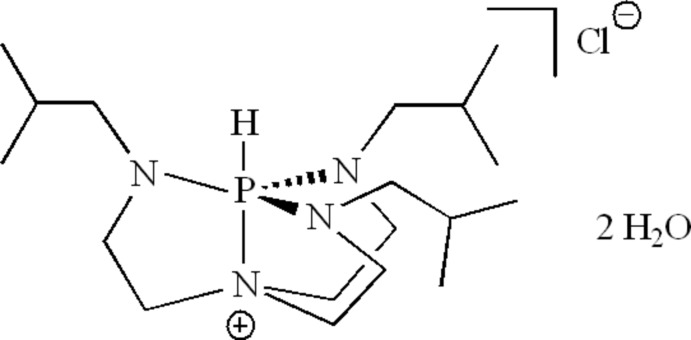


## Experimental

### 

#### Crystal data

C_18_H_40_N_4_P^+^·Cl^−^·2H_2_O
*M* *_r_* = 414.99
Triclinic, 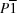

*a* = 10.0945 (19) Å
*b* = 15.759 (3) Å
*c* = 16.122 (3) Å
α = 106.720 (8)°
β = 92.259 (8)°
γ = 90.616 (8)°
*V* = 2453.6 (8) Å^3^
*Z* = 4
Mo *K*α radiation
μ = 0.24 mm^−1^
*T* = 296 K
0.20 × 0.15 × 0.14 mm

#### Data collection

Bruker APEXII CCD diffractometer
Absorption correction: multi-scan (*SADABS*; Bruker, 2004 ▶) *T*_min_ = 0.954, *T*_max_ = 0.967
35181 measured reflections
9856 independent reflections
5364 reflections with *I* > 2σ(*I*)
*R*_int_ = 0.063

#### Refinement

*R*[*F* ^2^ > 2σ(*F* ^2^)] = 0.068
*wR*(*F* ^2^) = 0.212
*S* = 1.01
9856 reflections
568 parameters
18 restraints
H atoms treated by a mixture of independent and constrained refinement
Δρ_max_ = 0.39 e Å^−3^
Δρ_min_ = −0.31 e Å^−3^

### 

Data collection: *APEX2* (Bruker, 2004 ▶); cell refinement: *SAINT* (Bruker, 2004 ▶); data reduction: *SAINT*; program(s) used to solve structure: *SHELXS97* (Sheldrick, 2008 ▶); program(s) used to refine structure: *SHELXL97* (Sheldrick, 2008 ▶); molecular graphics: *SHELXTL* (Sheldrick, 2008 ▶); software used to prepare material for publication: *SHELXTL*.

## Supplementary Material

Crystal structure: contains datablock(s) I, global. DOI: 10.1107/S1600536812045618/ff2086sup1.cif

Structure factors: contains datablock(s) I. DOI: 10.1107/S1600536812045618/ff2086Isup3.hkl

Supplementary material file. DOI: 10.1107/S1600536812045618/ff2086Isup4.cdx

Supplementary material file. DOI: 10.1107/S1600536812045618/ff2086Isup4.cml

Additional supplementary materials:  crystallographic information; 3D view; checkCIF report

## Figures and Tables

**Table 1:** Hydrogen-bond geometry (Å, °)

*D*—H⋯*A*	*D*—H	H⋯*A*	*D*⋯*A*	*D*—H⋯*A*
O1—H101⋯Cl2^i^	0.84 (6)	2.61 (6)	3.426 (6)	164 (5)
O1—H102⋯Cl2^ii^	0.93 (9)	2.32 (9)	3.208 (6)	159 (7)
O2—H103⋯Cl2^iii^	0.85 (7)	2.60 (7)	3.412 (7)	161 (5)
O2—H104⋯Cl2^ii^	0.81 (8)	2.38 (9)	3.188 (6)	177 (8)
O3—H105⋯Cl1^iii^	0.86 (5)	2.45 (5)	3.263 (6)	159 (4)
O3—H106⋯Cl1^iv^	0.85 (4)	2.45 (4)	3.286 (6)	165 (4)
O4—H108⋯Cl1^iii^	0.73 (5)	2.56 (5)	3.286 (6)	175 (4)

Symmetry codes: (i) 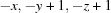; (ii) 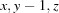; (iii) 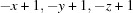; (iv) 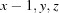.

**Fig. 1.**
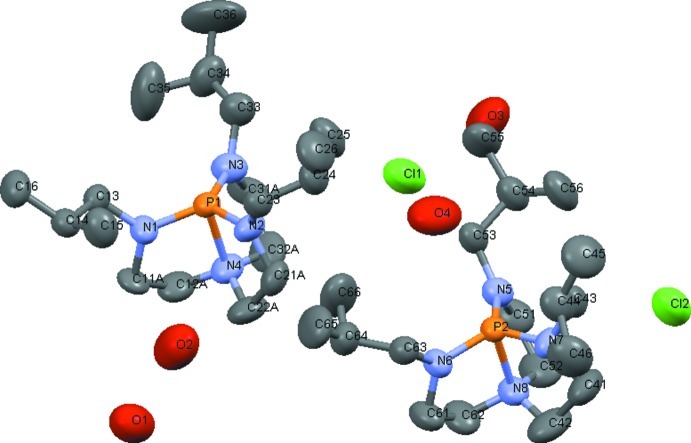
Molecular structure of the title compound, with the atom-numbering scheme. Displacement ellipsoids are drawn at the 50% probability level. H atoms are omitted for clarity.

## supplementary crystallographic information

### Comment

Nonionic proazaphosphatranes with stronger basicity than DBU (Tang *et al.*, 1993) have been used as stoichiometric bases and as catalysts in a wide range of organic reactions (Raders *et al.*, 2010; Verkade *et al.*, 2003; Zhou *et al.*, 2011). Unlike proazaphosphatranes, their protonated phosphonium salts known as azaphosphatranes with the five-membered tricyclic frameworks exist as solid states. Even though a lot of azaphosphatranes have appeared in the literature, only few examples of their solid state structures have been reported (Kingston *et al.*, 2005; Liu *et al.*, 1999; Liu *et al.*, 2000; Mohan *et al.*, 1996; Thirupathi *et al.*, 2003; Wroblewski *et al.*, 1995). In addition, the similar structure of the title complex (I) with four chloroform molecules in the monoclinic unit was reported in the literature; however, no crystallographic data and parameters were provided (Kisanga *et al.*, 2001). Herein, we report the X-ray structure of the title complex (I).

The title compound (I) could be isolated in more than 90% yield *via* the reaction of (i-BuNHCH_2_CH_2_)_3_N with ClP(NMe_2_)_2_, prepared *in situ* in acetonitrile by the slow addition of 1 equivalent of PCl_3_ to 2 equivalents of P(NMe_2_)_3_. In (I) (Fig. 1), the nearly ideal trigonal bipyramidal geometry around the phosphorus atom is confirmed by the sum of the N_eq_—P—N_eq_ angle of 358.8 ° and the average N_eq_—P—N_ax_ angle value of 86.3 °. The axial transannular P—N distance of 1.973 (2) Å and the average equatorial P—N bond distance of 1.664 (3) Å are in the range of typical values determined on azaphosphatranes derivatives (Kingston *et al.*, 2005; Liu *et al.*, 1999; Liu *et al.*, 2000; Mohan *et al.*, 1996; Thirupathi *et al.*, 2003; Wroblewski *et al.*, 1995).

### Experimental

The title compound (I) could be isolated in more than 90% yield *via* the reaction of (i-BuNHCH_2_CH_2_)_3_N with ClP(NMe_2_)_2_, prepared *in situ* in acetonitrile by the slow addition of 1 equivalent of PCl_3_ to 2 equivalents of P(NMe_2_)_3_. The crystal was obtained by slow evaporation of solvent in refrigerator.

### Refinement

H atoms attached to C atoms were positioned geometrically and refined using a riding model, with C—H = 0.93–0.97 Å and with *U*_iso_(H) = 1.2 (1.5 for methyl groups) times *U*_eq_(C). The water H atoms were found in difference Fourier maps and refined freely.

### Crystal data

**Table:**

C_18_H_40_N_4_P^+^·Cl^−^·2H_2_O	*Z* = 4
*M**_r_* = 414.99	*F*(000) = 912
Triclinic, *P*1	*D*_x_ = 1.123 Mg m^−^^3^
*a* = 10.0945 (19) Å	Mo *K*α radiation, λ = 0.71073 Å
*b* = 15.759 (3) Å	Cell parameters from 4603 reflections
*c* = 16.122 (3) Å	θ = 2.1–19.9°
α = 106.720 (8)°	µ = 0.24 mm^−^^1^
β = 92.259 (8)°	*T* = 296 K
γ = 90.616 (8)°	Block, colourless
*V* = 2453.6 (8) Å^3^	0.20 × 0.15 × 0.14 mm

### Data collection

**Table:**

Bruker APEXII CCD diffractometer	9856 independent reflections
Radiation source: fine-focus sealed tube	5364 reflections with *I* > 2σ(*I*)
Graphite monochromator	*R*_int_ = 0.063
φ and ω scans	θ_max_ = 26.6°, θ_min_ = 1.4°
Absorption correction: multi-scan (*SADABS*; Bruker, 2004)	*h* = −12→12
*T*_min_ = 0.954, *T*_max_ = 0.967	*k* = −19→18
35181 measured reflections	*l* = −19→19

### Refinement

**Table:**

Refinement on *F*^2^	Primary atom site location: structure-invariant direct methods
Least-squares matrix: full	Secondary atom site location: difference Fourier map
*R*[*F*^2^ > 2σ(*F*^2^)] = 0.068	Hydrogen site location: inferred from neighbouring sites
*wR*(*F*^2^) = 0.212	H atoms treated by a mixture of independent and constrained refinement
*S* = 1.01	*w* = 1/[σ^2^(*F*_o_^2^) + (0.1089*P*)^2^ + 0.247*P*] where *P* = (*F*_o_^2^ + 2*F*_c_^2^)/3
9856 reflections	(Δ/σ)_max_ = 0.011
568 parameters	Δρ_max_ = 0.39 e Å^−^^3^
18 restraints	Δρ_min_ = −0.31 e Å^−^^3^

### Special details

**Table:**

Geometry. All e.s.d.'s (except the e.s.d. in the dihedral angle between two l.s. planes) are estimated using the full covariance matrix. The cell e.s.d.'s are taken into account individually in the estimation of e.s.d.'s in distances, angles and torsion angles; correlations between e.s.d.'s in cell parameters are only used when they are defined by crystal symmetry. An approximate (isotropic) treatment of cell e.s.d.'s is used for estimating e.s.d.'s involving l.s. planes.
Refinement. Refinement of *F*^2^ against ALL reflections. The weighted *R*-factor *wR* and goodness of fit *S* are based on *F*^2^, conventional *R*-factors *R* are based on *F*, with *F* set to zero for negative *F*^2^. The threshold expression of *F*^2^ > σ(*F*^2^) is used only for calculating *R*-factors(gt) *etc*. and is not relevant to the choice of reflections for refinement. *R*-factors based on *F*^2^ are statistically about twice as large as those based on *F*, and *R*- factors based on ALL data will be even larger.

### Fractional atomic coordinates and isotropic or equivalent isotropic displacement parameters (Å^2^)

**Table:**

	*x*	*y*	*z*	*U*_iso_*/*U*_eq_	Occ. (<1)
P1	0.47871 (8)	0.18003 (5)	0.25193 (4)	0.0476 (2)	
H1	0.5165	0.1643	0.1863	0.057*	
N1	0.4185 (3)	0.07779 (17)	0.23900 (15)	0.0595 (7)	
N2	0.3761 (3)	0.25637 (18)	0.23615 (16)	0.0628 (7)	
N3	0.6278 (3)	0.2097 (2)	0.29888 (15)	0.0680 (8)	
N4	0.4135 (3)	0.20879 (19)	0.37035 (15)	0.0666 (8)	
C13	0.4487 (3)	0.00250 (19)	0.16562 (18)	0.0547 (8)	
H13A	0.4929	−0.0415	0.1875	0.066*	
H13B	0.5104	0.0224	0.1301	0.066*	
C14	0.3291 (3)	−0.0414 (2)	0.10863 (19)	0.0589 (8)	
H14	0.2676	−0.0616	0.1449	0.071*	
C15	0.2569 (4)	0.0221 (3)	0.0690 (3)	0.0878 (12)	
H15A	0.3168	0.0452	0.0357	0.132*	
H15B	0.2238	0.0700	0.1143	0.132*	
H15C	0.1841	−0.0086	0.0320	0.132*	
C16	0.3732 (4)	−0.1225 (2)	0.0384 (2)	0.0838 (11)	
H16A	0.4321	−0.1041	0.0012	0.126*	
H16B	0.2969	−0.1526	0.0048	0.126*	
H16C	0.4183	−0.1620	0.0650	0.126*	
C23	0.3569 (3)	0.2767 (2)	0.15394 (19)	0.0576 (8)	
H23A	0.2631	0.2706	0.1373	0.069*	
H23B	0.4027	0.2331	0.1100	0.069*	
C24	0.4048 (4)	0.3687 (2)	0.1538 (2)	0.0715 (10)	
H24	0.3522	0.4127	0.1940	0.086*	
C25	0.5489 (4)	0.3849 (3)	0.1837 (3)	0.1022 (14)	
H25A	0.6015	0.3402	0.1469	0.153*	
H25B	0.5595	0.3824	0.2424	0.153*	
H25C	0.5771	0.4423	0.1808	0.153*	
C26	0.3807 (5)	0.3789 (3)	0.0633 (3)	0.1052 (15)	
H26A	0.4367	0.3394	0.0237	0.158*	
H26B	0.4008	0.4389	0.0643	0.158*	
H26C	0.2895	0.3647	0.0449	0.158*	
C33	0.7437 (3)	0.2225 (3)	0.2514 (2)	0.0786 (11)	
H33A	0.7708	0.2845	0.2715	0.094*	
H33B	0.7171	0.2094	0.1905	0.094*	
C34	0.8623 (4)	0.1676 (3)	0.2595 (3)	0.0949 (14)	
H34	0.8966	0.1865	0.3198	0.114*	
C35	0.8185 (6)	0.0707 (3)	0.2372 (4)	0.153 (2)	
H35A	0.7853	0.0509	0.1781	0.230*	
H35B	0.7498	0.0643	0.2747	0.230*	
H35C	0.8927	0.0357	0.2449	0.230*	
C36	0.9706 (5)	0.1782 (5)	0.2028 (4)	0.187 (3)	
H36A	0.9395	0.1579	0.1432	0.280*	
H36B	1.0452	0.1441	0.2118	0.280*	
H36C	0.9970	0.2396	0.2169	0.280*	
C11A	0.3519 (12)	0.0572 (11)	0.3116 (10)	0.064 (4)	0.578 (9)
H11A	0.2566	0.0625	0.3054	0.077*	0.578 (9)
H11B	0.3710	−0.0026	0.3128	0.077*	0.578 (9)
C12A	0.4050 (11)	0.1221 (5)	0.3914 (4)	0.082 (3)	0.578 (9)
H12A	0.4919	0.1051	0.4079	0.098*	0.578 (9)
H12B	0.3464	0.1263	0.4386	0.098*	0.578 (9)
C21A	0.2888 (16)	0.2973 (9)	0.3003 (10)	0.069 (4)	0.578 (9)
H21A	0.2020	0.3007	0.2735	0.083*	0.578 (9)
H21B	0.3206	0.3572	0.3293	0.083*	0.578 (9)
C22A	0.2774 (8)	0.2474 (6)	0.3645 (4)	0.085 (3)	0.578 (9)
H22A	0.2101	0.2007	0.3454	0.102*	0.578 (9)
H22B	0.2546	0.2864	0.4203	0.102*	0.578 (9)
C31A	0.6527 (14)	0.2340 (12)	0.3908 (10)	0.090 (6)	0.578 (9)
H31A	0.7204	0.2805	0.4096	0.108*	0.578 (9)
H31B	0.6792	0.1835	0.4100	0.108*	0.578 (9)
C32A	0.5063 (9)	0.2696 (7)	0.4264 (4)	0.095 (3)	0.578 (9)
H32A	0.4984	0.2691	0.4860	0.114*	0.578 (9)
H32B	0.4926	0.3293	0.4229	0.114*	0.578 (9)
C11B	0.352 (3)	0.0518 (19)	0.3059 (18)	0.128 (11)	0.422 (9)
H11C	0.4104	0.0193	0.3342	0.153*	0.422 (9)
H11D	0.2736	0.0152	0.2821	0.153*	0.422 (9)
C12B	0.3107 (14)	0.1463 (8)	0.3741 (7)	0.089 (4)	0.422 (9)
H12C	0.2252	0.1649	0.3570	0.106*	0.422 (9)
H12D	0.3065	0.1408	0.4324	0.106*	0.422 (9)
C21B	0.311 (2)	0.3230 (14)	0.3157 (14)	0.076 (6)	0.422 (9)
H21C	0.3266	0.3838	0.3156	0.091*	0.422 (9)
H21D	0.2160	0.3117	0.3142	0.091*	0.422 (9)
C22B	0.3783 (11)	0.3057 (7)	0.3948 (5)	0.077 (4)	0.422 (9)
H22C	0.4577	0.3427	0.4125	0.093*	0.422 (9)
H22D	0.3191	0.3191	0.4426	0.093*	0.422 (9)
C31B	0.638 (3)	0.2444 (19)	0.3975 (16)	0.116 (10)	0.422 (9)
H31C	0.7253	0.2328	0.4193	0.140*	0.422 (9)
H31D	0.6244	0.3077	0.4165	0.140*	0.422 (9)
C32B	0.5336 (13)	0.1961 (11)	0.4291 (6)	0.095 (4)	0.422 (9)
H32C	0.5190	0.2230	0.4901	0.113*	0.422 (9)
H32D	0.5538	0.1341	0.4192	0.113*	0.422 (9)
P2	0.00164 (8)	0.67818 (5)	0.25974 (4)	0.0463 (2)	
H2	0.0358	0.6634	0.1932	0.056*	
N5	0.1546 (3)	0.67602 (17)	0.30179 (15)	0.0571 (7)	
N6	−0.0976 (3)	0.58841 (17)	0.23778 (14)	0.0585 (7)	
N7	−0.0637 (3)	0.77358 (17)	0.25760 (15)	0.0644 (7)	
N8	−0.0565 (3)	0.70466 (19)	0.37922 (15)	0.0634 (7)	
C41	−0.1248 (5)	0.8303 (3)	0.3349 (3)	0.0971 (14)	
H41A	−0.2028	0.8582	0.3186	0.116*	
H41B	−0.0625	0.8761	0.3677	0.116*	
C42	−0.1624 (5)	0.7683 (3)	0.3881 (3)	0.1092 (16)	
H42A	−0.1703	0.8014	0.4484	0.131*	
H42B	−0.2464	0.7380	0.3663	0.131*	
C43	−0.0232 (3)	0.8174 (2)	0.1939 (2)	0.0599 (8)	
H43A	0.0653	0.7987	0.1768	0.072*	
H43B	−0.0184	0.8808	0.2217	0.072*	
C44	−0.1139 (4)	0.7998 (3)	0.1120 (2)	0.0772 (11)	
H44	−0.1135	0.7358	0.0837	0.093*	
C45	−0.0542 (5)	0.8441 (3)	0.0499 (3)	0.1091 (15)	
H45A	−0.1088	0.8311	−0.0025	0.164*	
H45B	0.0333	0.8223	0.0367	0.164*	
H45C	−0.0491	0.9071	0.0762	0.164*	
C46	−0.2533 (4)	0.8234 (3)	0.1279 (3)	0.1177 (16)	
H46A	−0.2581	0.8856	0.1570	0.177*	
H46B	−0.2894	0.7902	0.1635	0.177*	
H46C	−0.3034	0.8098	0.0737	0.177*	
C51	0.1787 (4)	0.6952 (3)	0.3955 (2)	0.0859 (12)	
H51A	0.2580	0.7322	0.4142	0.103*	
H51B	0.1907	0.6407	0.4111	0.103*	
C52	0.0604 (5)	0.7423 (3)	0.4373 (2)	0.1013 (15)	
H52A	0.0507	0.7333	0.4938	0.122*	
H52B	0.0696	0.8054	0.4446	0.122*	
C53	0.2650 (3)	0.6351 (2)	0.2489 (2)	0.0605 (8)	
H53A	0.2335	0.6145	0.1887	0.073*	
H53B	0.2924	0.5836	0.2662	0.073*	
C54	0.3857 (4)	0.6966 (2)	0.2560 (2)	0.0732 (10)	
H54	0.4206	0.7135	0.3162	0.088*	
C55	0.4924 (4)	0.6446 (3)	0.1989 (4)	0.1183 (17)	
H55A	0.4651	0.6338	0.1389	0.177*	
H55B	0.5048	0.5891	0.2112	0.177*	
H55C	0.5743	0.6783	0.2106	0.177*	
C56	0.3497 (4)	0.7796 (3)	0.2337 (3)	0.0973 (13)	
H56A	0.4271	0.8173	0.2404	0.146*	
H56B	0.2835	0.8100	0.2716	0.146*	
H56C	0.3155	0.7647	0.1747	0.146*	
C61	−0.1721 (4)	0.5683 (3)	0.3062 (2)	0.0801 (11)	
H61A	−0.1743	0.5050	0.2991	0.096*	
H61B	−0.2625	0.5884	0.3051	0.096*	
C62	−0.0976 (5)	0.6183 (3)	0.3917 (2)	0.0916 (13)	
H62A	−0.1551	0.6273	0.4402	0.110*	
H62B	−0.0208	0.5858	0.4023	0.110*	
C63	−0.1421 (3)	0.5396 (2)	0.14950 (18)	0.0564 (8)	
H63A	−0.1010	0.5667	0.1098	0.068*	
H63B	−0.2372	0.5460	0.1436	0.068*	
C64	−0.1124 (4)	0.4416 (2)	0.1222 (2)	0.0792 (11)	
H64	−0.1614	0.4146	0.1597	0.095*	
C65	−0.1675 (5)	0.4001 (3)	0.0296 (3)	0.1206 (18)	
H65A	−0.2618	0.4071	0.0273	0.181*	
H65B	−0.1480	0.3381	0.0116	0.181*	
H65C	−0.1274	0.4288	−0.0085	0.181*	
C66	0.0313 (5)	0.4249 (3)	0.1354 (4)	0.136 (2)	
H66A	0.0831	0.4526	0.1013	0.203*	
H66B	0.0456	0.3622	0.1178	0.203*	
H66C	0.0576	0.4491	0.1956	0.203*	
Cl1	0.74844 (12)	0.48539 (9)	0.49895 (10)	0.1183 (5)	
Cl2	0.24967 (11)	0.95105 (8)	0.49482 (9)	0.1136 (4)	
O1	0.0445 (6)	0.0588 (3)	0.4090 (3)	0.1250 (13)	
H101	−0.033 (6)	0.050 (4)	0.422 (4)	0.14 (3)*	
H102	0.086 (8)	0.016 (6)	0.430 (5)	0.25 (4)*	
O2	0.4520 (7)	0.1069 (3)	0.5950 (3)	0.1382 (16)	
H103	0.530 (7)	0.088 (4)	0.584 (4)	0.16 (3)*	
H104	0.403 (8)	0.066 (6)	0.570 (5)	0.24 (5)*	
O3	−0.0019 (6)	0.6098 (3)	0.6065 (3)	0.1294 (15)	
H105	0.076 (5)	0.593 (3)	0.591 (3)	0.098 (17)*	
H106	−0.072 (4)	0.578 (3)	0.588 (3)	0.091 (17)*	
O4	0.5064 (6)	0.5514 (4)	0.3944 (3)	0.1389 (17)	
H107	0.551 (6)	0.506 (4)	0.414 (4)	0.16 (3)*	
H108	0.448 (5)	0.541 (3)	0.415 (3)	0.086 (18)*	

### Atomic displacement parameters (Å^2^)

**Table:**

	*U*^11^	*U*^22^	*U*^33^	*U*^12^	*U*^13^	*U*^23^
P1	0.0493 (5)	0.0529 (5)	0.0378 (4)	0.0011 (4)	0.0066 (3)	0.0077 (3)
N1	0.0780 (19)	0.0541 (16)	0.0458 (13)	−0.0067 (14)	0.0140 (13)	0.0121 (12)
N2	0.0663 (18)	0.0635 (18)	0.0593 (15)	0.0197 (15)	0.0190 (13)	0.0157 (13)
N3	0.0575 (18)	0.091 (2)	0.0497 (14)	−0.0071 (16)	−0.0033 (13)	0.0116 (14)
N4	0.081 (2)	0.074 (2)	0.0421 (13)	0.0076 (17)	0.0139 (13)	0.0101 (13)
C13	0.061 (2)	0.0480 (18)	0.0553 (17)	0.0068 (15)	0.0082 (14)	0.0145 (14)
C14	0.061 (2)	0.059 (2)	0.0570 (17)	−0.0051 (16)	0.0069 (15)	0.0158 (15)
C15	0.080 (3)	0.083 (3)	0.098 (3)	−0.005 (2)	−0.020 (2)	0.027 (2)
C16	0.108 (3)	0.065 (2)	0.069 (2)	−0.006 (2)	0.008 (2)	0.0044 (18)
C23	0.052 (2)	0.057 (2)	0.0640 (18)	0.0018 (16)	−0.0011 (15)	0.0186 (15)
C24	0.071 (3)	0.057 (2)	0.091 (2)	0.0041 (18)	0.0058 (19)	0.0276 (18)
C25	0.077 (3)	0.082 (3)	0.156 (4)	−0.017 (2)	−0.006 (3)	0.050 (3)
C26	0.129 (4)	0.093 (3)	0.114 (3)	0.005 (3)	0.007 (3)	0.061 (3)
C33	0.059 (2)	0.088 (3)	0.082 (2)	−0.002 (2)	−0.0052 (19)	0.016 (2)
C34	0.081 (3)	0.088 (3)	0.102 (3)	0.025 (2)	−0.013 (2)	0.006 (2)
C35	0.161 (5)	0.079 (4)	0.191 (6)	0.024 (4)	−0.041 (4)	−0.001 (3)
C36	0.060 (3)	0.286 (9)	0.191 (6)	0.002 (4)	0.037 (4)	0.028 (6)
C11A	0.077 (6)	0.070 (7)	0.056 (7)	−0.012 (5)	0.022 (5)	0.033 (6)
C12A	0.102 (7)	0.095 (6)	0.055 (4)	0.014 (5)	0.030 (4)	0.029 (4)
C21A	0.084 (7)	0.052 (8)	0.065 (6)	0.019 (6)	0.003 (4)	0.006 (6)
C22A	0.083 (6)	0.105 (7)	0.065 (4)	0.025 (5)	0.034 (4)	0.018 (4)
C31A	0.073 (8)	0.127 (10)	0.049 (6)	−0.049 (8)	0.005 (5)	−0.006 (6)
C32A	0.134 (9)	0.094 (6)	0.042 (3)	−0.011 (6)	−0.004 (4)	−0.002 (4)
C11B	0.21 (2)	0.084 (14)	0.069 (13)	−0.002 (14)	0.044 (13)	−0.011 (10)
C12B	0.096 (9)	0.084 (8)	0.079 (7)	−0.023 (7)	0.051 (7)	0.008 (6)
C21B	0.086 (10)	0.055 (10)	0.072 (9)	0.006 (7)	0.051 (8)	−0.012 (7)
C22B	0.079 (8)	0.081 (7)	0.057 (5)	0.001 (6)	0.026 (5)	−0.008 (4)
C31B	0.115 (16)	0.165 (18)	0.054 (11)	0.067 (14)	−0.036 (10)	0.008 (10)
C32B	0.106 (9)	0.136 (12)	0.042 (5)	0.012 (9)	0.014 (5)	0.026 (6)
P2	0.0527 (5)	0.0490 (5)	0.0377 (4)	−0.0003 (4)	0.0066 (3)	0.0127 (3)
N5	0.0556 (17)	0.0674 (17)	0.0497 (13)	−0.0043 (14)	−0.0030 (12)	0.0198 (12)
N6	0.0673 (17)	0.0603 (17)	0.0448 (13)	−0.0160 (14)	0.0048 (12)	0.0103 (12)
N7	0.083 (2)	0.0593 (17)	0.0530 (14)	0.0176 (15)	0.0152 (13)	0.0167 (12)
N8	0.075 (2)	0.0684 (18)	0.0432 (13)	−0.0108 (16)	0.0101 (13)	0.0100 (12)
C41	0.129 (4)	0.078 (3)	0.082 (3)	0.036 (3)	0.041 (3)	0.014 (2)
C42	0.128 (4)	0.128 (4)	0.071 (2)	0.042 (3)	0.043 (2)	0.021 (2)
C43	0.059 (2)	0.0486 (19)	0.074 (2)	0.0025 (16)	−0.0026 (16)	0.0218 (16)
C44	0.069 (3)	0.084 (3)	0.089 (2)	0.006 (2)	−0.008 (2)	0.045 (2)
C45	0.122 (4)	0.125 (4)	0.102 (3)	0.013 (3)	−0.004 (3)	0.068 (3)
C46	0.076 (3)	0.136 (4)	0.146 (4)	−0.001 (3)	−0.015 (3)	0.052 (3)
C51	0.086 (3)	0.116 (3)	0.058 (2)	−0.016 (3)	−0.011 (2)	0.032 (2)
C52	0.116 (4)	0.131 (4)	0.0467 (19)	−0.021 (3)	0.002 (2)	0.011 (2)
C53	0.051 (2)	0.056 (2)	0.079 (2)	−0.0014 (16)	−0.0035 (16)	0.0261 (16)
C54	0.063 (2)	0.070 (2)	0.093 (2)	−0.0080 (19)	−0.0074 (19)	0.034 (2)
C55	0.060 (3)	0.098 (3)	0.195 (5)	0.003 (3)	0.030 (3)	0.037 (3)
C56	0.084 (3)	0.078 (3)	0.145 (4)	−0.015 (2)	−0.005 (3)	0.059 (3)
C61	0.089 (3)	0.088 (3)	0.0593 (19)	−0.028 (2)	0.0184 (19)	0.0147 (18)
C62	0.125 (4)	0.096 (3)	0.058 (2)	−0.022 (3)	0.023 (2)	0.027 (2)
C63	0.0521 (19)	0.062 (2)	0.0509 (16)	−0.0023 (16)	0.0014 (14)	0.0099 (14)
C64	0.081 (3)	0.060 (2)	0.088 (2)	0.001 (2)	0.015 (2)	0.0070 (19)
C65	0.154 (5)	0.092 (3)	0.083 (3)	−0.022 (3)	0.017 (3)	−0.027 (2)
C66	0.094 (4)	0.088 (4)	0.215 (6)	0.031 (3)	0.023 (4)	0.024 (3)
Cl1	0.0861 (8)	0.1121 (10)	0.1764 (13)	0.0095 (7)	0.0333 (8)	0.0692 (9)
Cl2	0.0769 (8)	0.1007 (9)	0.1633 (12)	−0.0105 (6)	0.0060 (7)	0.0387 (8)
O1	0.114 (3)	0.133 (3)	0.139 (3)	0.003 (3)	0.023 (3)	0.055 (3)
O2	0.125 (4)	0.140 (4)	0.121 (3)	0.002 (3)	−0.002 (3)	−0.007 (2)
O3	0.127 (4)	0.128 (3)	0.112 (3)	0.022 (3)	0.012 (3)	−0.001 (2)
O4	0.136 (4)	0.192 (5)	0.109 (3)	0.017 (3)	0.030 (3)	0.071 (3)

### Geometric parameters (Å, º)

**Table:**

P1—N3	1.658 (3)	C22B—H22D	0.9700
P1—N2	1.664 (3)	C31B—C32B	1.48 (3)
P1—N1	1.669 (3)	C31B—H31C	0.9700
P1—N4	1.973 (2)	C31B—H31D	0.9700
P1—H1	1.1000	C32B—H32C	0.9700
N1—C11B	1.45 (3)	C32B—H32D	0.9700
N1—C13	1.459 (4)	P2—N7	1.657 (3)
N1—C11A	1.484 (12)	P2—N5	1.666 (3)
N2—C21A	1.400 (16)	P2—N6	1.669 (3)
N2—C23	1.457 (4)	P2—N8	1.965 (2)
N2—C21B	1.577 (19)	P2—H2	1.1000
N3—C31A	1.431 (15)	N5—C51	1.463 (4)
N3—C33	1.466 (4)	N5—C53	1.470 (4)
N3—C31B	1.52 (2)	N6—C63	1.460 (3)
N4—C32A	1.424 (8)	N6—C61	1.465 (4)
N4—C12B	1.438 (10)	N7—C43	1.460 (4)
N4—C12A	1.502 (8)	N7—C41	1.470 (4)
N4—C22B	1.513 (10)	N8—C42	1.456 (5)
N4—C22A	1.519 (7)	N8—C52	1.483 (5)
N4—C32B	1.559 (12)	N8—C62	1.489 (5)
C13—C14	1.519 (4)	C41—C42	1.528 (6)
C13—H13A	0.9700	C41—H41A	0.9700
C13—H13B	0.9700	C41—H41B	0.9700
C14—C15	1.513 (5)	C42—H42A	0.9700
C14—C16	1.528 (4)	C42—H42B	0.9700
C14—H14	0.9800	C43—C44	1.533 (4)
C15—H15A	0.9600	C43—H43A	0.9700
C15—H15B	0.9600	C43—H43B	0.9700
C15—H15C	0.9600	C44—C46	1.473 (5)
C16—H16A	0.9600	C44—C45	1.515 (5)
C16—H16B	0.9600	C44—H44	0.9800
C16—H16C	0.9600	C45—H45A	0.9600
C23—C24	1.524 (4)	C45—H45B	0.9600
C23—H23A	0.9700	C45—H45C	0.9600
C23—H23B	0.9700	C46—H46A	0.9600
C24—C25	1.509 (5)	C46—H46B	0.9600
C24—C26	1.523 (5)	C46—H46C	0.9600
C24—H24	0.9800	C51—C52	1.493 (6)
C25—H25A	0.9600	C51—H51A	0.9700
C25—H25B	0.9600	C51—H51B	0.9700
C25—H25C	0.9600	C52—H52A	0.9700
C26—H26A	0.9600	C52—H52B	0.9700
C26—H26B	0.9600	C53—C54	1.530 (5)
C26—H26C	0.9600	C53—H53A	0.9700
C33—C34	1.508 (5)	C53—H53B	0.9700
C33—H33A	0.9700	C54—C56	1.497 (5)
C33—H33B	0.9700	C54—C55	1.530 (5)
C34—C36	1.491 (7)	C54—H54	0.9800
C34—C35	1.521 (6)	C55—H55A	0.9600
C34—H34	0.9800	C55—H55B	0.9600
C35—H35A	0.9600	C55—H55C	0.9600
C35—H35B	0.9600	C56—H56A	0.9600
C35—H35C	0.9600	C56—H56B	0.9600
C36—H36A	0.9600	C56—H56C	0.9600
C36—H36B	0.9600	C61—C62	1.538 (5)
C36—H36C	0.9600	C61—H61A	0.9700
C11A—C12A	1.470 (18)	C61—H61B	0.9700
C11A—H11A	0.9700	C62—H62A	0.9700
C11A—H11B	0.9700	C62—H62B	0.9700
C12A—H12A	0.9700	C63—C64	1.515 (4)
C12A—H12B	0.9700	C63—H63A	0.9700
C21A—C22A	1.476 (17)	C63—H63B	0.9700
C21A—H21A	0.9700	C64—C66	1.495 (6)
C21A—H21B	0.9700	C64—C65	1.527 (5)
C22A—H22A	0.9700	C64—H64	0.9800
C22A—H22B	0.9700	C65—H65A	0.9600
C31A—C32A	1.65 (2)	C65—H65B	0.9600
C31A—H31A	0.9700	C65—H65C	0.9600
C31A—H31B	0.9700	C66—H66A	0.9600
C32A—H32A	0.9700	C66—H66B	0.9600
C32A—H32B	0.9700	C66—H66C	0.9600
C11B—C12B	1.64 (3)	O1—H101	0.84 (6)
C11B—H11C	0.9700	O1—H102	0.94 (9)
C11B—H11D	0.9700	O2—H103	0.85 (7)
C12B—H12C	0.9700	O2—H104	0.80 (8)
C12B—H12D	0.9700	O3—H105	0.85 (4)
C21B—C22B	1.52 (3)	O3—H106	0.86 (4)
C21B—H21C	0.9700	O4—H107	0.97 (6)
C21B—H21D	0.9700	O4—H108	0.73 (4)
C22B—H22C	0.9700		
			
N3—P1—N2	120.17 (15)	C22B—C21B—H21C	110.8
N3—P1—N1	120.09 (15)	N2—C21B—H21C	110.8
N2—P1—N1	118.53 (15)	C22B—C21B—H21D	110.8
N3—P1—N4	86.27 (13)	N2—C21B—H21D	110.8
N2—P1—N4	86.52 (12)	H21C—C21B—H21D	108.9
N1—P1—N4	86.24 (12)	N4—C22B—C21B	107.5 (9)
N3—P1—H1	92.9	N4—C22B—H22C	110.2
N2—P1—H1	93.9	C21B—C22B—H22C	110.2
N1—P1—H1	94.2	N4—C22B—H22D	110.2
N4—P1—H1	179.2	C21B—C22B—H22D	110.2
C11B—N1—C13	112.8 (11)	H22C—C22B—H22D	108.5
C13—N1—C11A	116.6 (7)	C32B—C31B—N3	105.9 (16)
C11B—N1—P1	123.3 (11)	C32B—C31B—H31C	110.6
C13—N1—P1	123.0 (2)	N3—C31B—H31C	110.6
C11A—N1—P1	119.5 (7)	C32B—C31B—H31D	110.6
C21A—N2—C23	115.4 (6)	N3—C31B—H31D	110.6
C23—N2—C21B	115.1 (9)	H31C—C31B—H31D	108.7
C21A—N2—P1	120.0 (6)	C31B—C32B—N4	99.7 (10)
C23—N2—P1	124.0 (2)	C31B—C32B—H32C	111.8
C21B—N2—P1	120.2 (9)	N4—C32B—H32C	111.8
C31A—N3—C33	113.3 (6)	C31B—C32B—H32D	111.8
C33—N3—C31B	117.4 (11)	N4—C32B—H32D	111.8
C31A—N3—P1	123.0 (6)	H32C—C32B—H32D	109.6
C33—N3—P1	123.4 (2)	N7—P2—N5	120.29 (14)
C31B—N3—P1	117.9 (10)	N7—P2—N6	118.48 (15)
C32A—N4—C12B	139.9 (6)	N5—P2—N6	120.12 (14)
C32A—N4—C12A	112.5 (6)	N7—P2—N8	86.81 (12)
C12B—N4—C22B	117.2 (7)	N5—P2—N8	86.24 (12)
C12A—N4—C22B	147.4 (5)	N6—P2—N8	86.40 (11)
C32A—N4—C22A	113.5 (5)	N7—P2—H2	93.6
C12A—N4—C22A	111.7 (5)	N5—P2—H2	92.9
C12B—N4—C32B	107.8 (8)	N6—P2—H2	94.1
C22B—N4—C32B	109.4 (7)	N8—P2—H2	179.1
C22A—N4—C32B	147.8 (5)	C51—N5—C53	115.2 (3)
C32A—N4—P1	107.7 (4)	C51—N5—P2	120.7 (2)
C12B—N4—P1	109.9 (4)	C53—N5—P2	122.48 (19)
C12A—N4—P1	105.6 (3)	C63—N6—C61	115.0 (3)
C22B—N4—P1	106.3 (4)	C63—N6—P2	122.5 (2)
C22A—N4—P1	105.3 (3)	C61—N6—P2	120.7 (2)
C32B—N4—P1	105.7 (4)	C43—N7—C41	116.0 (3)
N1—C13—C14	114.8 (3)	C43—N7—P2	120.5 (2)
N1—C13—H13A	108.6	C41—N7—P2	120.2 (2)
C14—C13—H13A	108.6	C42—N8—C52	111.8 (3)
N1—C13—H13B	108.6	C42—N8—C62	114.4 (3)
C14—C13—H13B	108.6	C52—N8—C62	109.9 (3)
H13A—C13—H13B	107.5	C42—N8—P2	107.1 (2)
C15—C14—C13	112.1 (3)	C52—N8—P2	106.9 (2)
C15—C14—C16	110.9 (3)	C62—N8—P2	106.33 (19)
C13—C14—C16	109.5 (3)	N7—C41—C42	105.4 (3)
C15—C14—H14	108.1	N7—C41—H41A	110.7
C13—C14—H14	108.1	C42—C41—H41A	110.7
C16—C14—H14	108.1	N7—C41—H41B	110.7
C14—C15—H15A	109.5	C42—C41—H41B	110.7
C14—C15—H15B	109.5	H41A—C41—H41B	108.8
H15A—C15—H15B	109.5	N8—C42—C41	106.4 (3)
C14—C15—H15C	109.5	N8—C42—H42A	110.5
H15A—C15—H15C	109.5	C41—C42—H42A	110.5
H15B—C15—H15C	109.5	N8—C42—H42B	110.5
C14—C16—H16A	109.5	C41—C42—H42B	110.5
C14—C16—H16B	109.5	H42A—C42—H42B	108.6
H16A—C16—H16B	109.5	N7—C43—C44	115.5 (3)
C14—C16—H16C	109.5	N7—C43—H43A	108.4
H16A—C16—H16C	109.5	C44—C43—H43A	108.4
H16B—C16—H16C	109.5	N7—C43—H43B	108.4
N2—C23—C24	115.5 (3)	C44—C43—H43B	108.4
N2—C23—H23A	108.4	H43A—C43—H43B	107.5
C24—C23—H23A	108.4	C46—C44—C45	112.2 (3)
N2—C23—H23B	108.4	C46—C44—C43	114.7 (3)
C24—C23—H23B	108.4	C45—C44—C43	109.3 (3)
H23A—C23—H23B	107.5	C46—C44—H44	106.7
C25—C24—C26	111.6 (4)	C45—C44—H44	106.7
C25—C24—C23	111.4 (3)	C43—C44—H44	106.7
C26—C24—C23	109.0 (3)	C44—C45—H45A	109.5
C25—C24—H24	108.3	C44—C45—H45B	109.5
C26—C24—H24	108.3	H45A—C45—H45B	109.5
C23—C24—H24	108.3	C44—C45—H45C	109.5
C24—C25—H25A	109.5	H45A—C45—H45C	109.5
C24—C25—H25B	109.5	H45B—C45—H45C	109.5
H25A—C25—H25B	109.5	C44—C46—H46A	109.5
C24—C25—H25C	109.5	C44—C46—H46B	109.5
H25A—C25—H25C	109.5	H46A—C46—H46B	109.5
H25B—C25—H25C	109.5	C44—C46—H46C	109.5
C24—C26—H26A	109.5	H46A—C46—H46C	109.5
C24—C26—H26B	109.5	H46B—C46—H46C	109.5
H26A—C26—H26B	109.5	N5—C51—C52	106.9 (3)
C24—C26—H26C	109.5	N5—C51—H51A	110.3
H26A—C26—H26C	109.5	C52—C51—H51A	110.3
H26B—C26—H26C	109.5	N5—C51—H51B	110.3
N3—C33—C34	115.9 (4)	C52—C51—H51B	110.3
N3—C33—H33A	108.3	H51A—C51—H51B	108.6
C34—C33—H33A	108.3	N8—C52—C51	106.9 (3)
N3—C33—H33B	108.3	N8—C52—H52A	110.3
C34—C33—H33B	108.3	C51—C52—H52A	110.3
H33A—C33—H33B	107.4	N8—C52—H52B	110.3
C36—C34—C33	112.4 (5)	C51—C52—H52B	110.3
C36—C34—C35	110.1 (5)	H52A—C52—H52B	108.6
C33—C34—C35	109.1 (4)	N5—C53—C54	114.4 (3)
C36—C34—H34	108.4	N5—C53—H53A	108.7
C33—C34—H34	108.4	C54—C53—H53A	108.7
C35—C34—H34	108.4	N5—C53—H53B	108.7
C34—C35—H35A	109.5	C54—C53—H53B	108.7
C34—C35—H35B	109.5	H53A—C53—H53B	107.6
H35A—C35—H35B	109.5	C56—C54—C53	111.7 (3)
C34—C35—H35C	109.5	C56—C54—C55	112.7 (4)
H35A—C35—H35C	109.5	C53—C54—C55	108.3 (3)
H35B—C35—H35C	109.5	C56—C54—H54	108.0
C34—C36—H36A	109.5	C53—C54—H54	108.0
C34—C36—H36B	109.5	C55—C54—H54	108.0
H36A—C36—H36B	109.5	C54—C55—H55A	109.5
C34—C36—H36C	109.5	C54—C55—H55B	109.5
H36A—C36—H36C	109.5	H55A—C55—H55B	109.5
H36B—C36—H36C	109.5	C54—C55—H55C	109.5
C12A—C11A—N1	106.2 (9)	H55A—C55—H55C	109.5
C12A—C11A—H11A	110.5	H55B—C55—H55C	109.5
N1—C11A—H11A	110.5	C54—C56—H56A	109.5
C12A—C11A—H11B	110.5	C54—C56—H56B	109.5
N1—C11A—H11B	110.5	H56A—C56—H56B	109.5
H11A—C11A—H11B	108.7	C54—C56—H56C	109.5
C11A—C12A—N4	105.6 (7)	H56A—C56—H56C	109.5
C11A—C12A—H12A	110.6	H56B—C56—H56C	109.5
N4—C12A—H12A	110.6	N6—C61—C62	105.4 (3)
C11A—C12A—H12B	110.6	N6—C61—H61A	110.7
N4—C12A—H12B	110.6	C62—C61—H61A	110.7
H12A—C12A—H12B	108.7	N6—C61—H61B	110.7
N2—C21A—C22A	111.0 (9)	C62—C61—H61B	110.7
N2—C21A—H21A	109.4	H61A—C61—H61B	108.8
C22A—C21A—H21A	109.4	N8—C62—C61	104.7 (3)
N2—C21A—H21B	109.4	N8—C62—H62A	110.8
C22A—C21A—H21B	109.4	C61—C62—H62A	110.8
H21A—C21A—H21B	108.0	N8—C62—H62B	110.8
C21A—C22A—N4	104.9 (8)	C61—C62—H62B	110.8
C21A—C22A—H22A	110.8	H62A—C62—H62B	108.9
N4—C22A—H22A	110.8	N6—C63—C64	115.6 (3)
C21A—C22A—H22B	110.8	N6—C63—H63A	108.4
N4—C22A—H22B	110.8	C64—C63—H63A	108.4
H22A—C22A—H22B	108.8	N6—C63—H63B	108.4
N3—C31A—C32A	101.6 (9)	C64—C63—H63B	108.4
N3—C31A—H31A	111.4	H63A—C63—H63B	107.5
C32A—C31A—H31A	111.4	C66—C64—C63	112.3 (3)
N3—C31A—H31B	111.4	C66—C64—C65	114.0 (4)
C32A—C31A—H31B	111.4	C63—C64—C65	109.1 (3)
H31A—C31A—H31B	109.3	C66—C64—H64	107.1
N4—C32A—C31A	104.6 (7)	C63—C64—H64	107.1
N4—C32A—H32A	110.8	C65—C64—H64	107.1
C31A—C32A—H32A	110.8	C64—C65—H65A	109.5
N4—C32A—H32B	110.8	C64—C65—H65B	109.5
C31A—C32A—H32B	110.8	H65A—C65—H65B	109.5
H32A—C32A—H32B	108.9	C64—C65—H65C	109.5
N1—C11B—C12B	104.1 (18)	H65A—C65—H65C	109.5
N1—C11B—H11C	110.9	H65B—C65—H65C	109.5
C12B—C11B—H11C	110.9	C64—C66—H66A	109.5
N1—C11B—H11D	110.9	C64—C66—H66B	109.5
C12B—C11B—H11D	110.9	H66A—C66—H66B	109.5
H11C—C11B—H11D	109.0	C64—C66—H66C	109.5
N4—C12B—C11B	105.4 (12)	H66A—C66—H66C	109.5
N4—C12B—H12C	110.7	H66B—C66—H66C	109.5
C11B—C12B—H12C	110.7	H101—O1—H102	98 (6)
N4—C12B—H12D	110.7	H103—O2—H104	105 (7)
C11B—C12B—H12D	110.7	H105—O3—H106	123 (5)
H12C—C12B—H12D	108.8	H107—O4—H108	86 (5)
C22B—C21B—N2	104.7 (14)		

### Hydrogen-bond geometry (Å, º)

**Table:**

*D*—H···*A*	*D*—H	H···*A*	*D*···*A*	*D*—H···*A*
O1—H101···Cl2^i^	0.84 (6)	2.61 (6)	3.426 (6)	164 (5)
O1—H102···Cl2^ii^	0.93 (9)	2.32 (9)	3.208 (6)	159 (7)
O2—H103···Cl2^iii^	0.85 (7)	2.60 (7)	3.412 (7)	161 (5)
O2—H104···Cl2^ii^	0.81 (8)	2.38 (9)	3.188 (6)	177 (8)
O3—H105···Cl1^iii^	0.86 (5)	2.45 (5)	3.263 (6)	159 (4)
O3—H106···Cl1^iv^	0.85 (4)	2.45 (4)	3.286 (6)	165 (4)
O4—H108···Cl1^iii^	0.73 (5)	2.56 (5)	3.286 (6)	175 (4)

Symmetry codes: (i) −*x*, −*y*+1, −*z*+1; (ii) *x*, *y*−1, *z*; (iii) −*x*+1, −*y*+1, −*z*+1; (iv) *x*−1, *y*, *z*.
